# Maternal and child under-nutrition in rural and urban communities of Lagos state, Nigeria: the relationship and risk factors

**DOI:** 10.1186/1756-0500-6-286

**Published:** 2013-07-23

**Authors:** Idowu O Senbanjo, Ibiyemi O Olayiwola, Wasiu A Afolabi, Olayinka C Senbanjo

**Affiliations:** 1 Department of Paediatrics and Child Health, Lagos State University College of Medicine, Ikeja, Lagos PMB 21266, Nigeria; 2 Department of Nutrition and Dietetics, College of Food Sciences and Human Ecology, Federal University of Agriculture Abeokuta, FUNAAB P.O Box 54, Alabata Road, Abeokuta, Nigeria; 3 Department of Obstetrics and Gynaecology, Lagos State University Teaching Hospital, No1-5, Oba Akinjobi Way, GRA, Ikeja, Lagos State, Nigeria; 4 Department of Paediatrics and Child Health, Paediatrics Gastroenterology/Hepatology/Nutrition Unit, Lagos State University Teaching Hospital, Ikeja, Lagos, Nigeria

**Keywords:** Mother, Children, Under-nutrition, Risk factors, Rural, Urban, Nigeria

## Abstract

**Background:**

Poor nutritional status of mothers has a direct and indirect consequence on their own health and that of their children. The objective of this study was to determine the relationship between nutritional status of mothers and their children and the risk factors for under-nutrition among mothers and children in rural and urban communities of Lagos State, Nigeria.

**Methods:**

This was a cross sectional survey conducted using the multistage random sampling technique. A total of 300 mother-child pairs were studied, consisting of 150 each from rural and urban communities. Under-nutrition in mothers and children was determined using standard criteria.

**Results:**

The prevalence of under-nutrition among mothers was significantly higher in rural than urban communities (10.7% vs. 2.7%, p = 0.014). The prevalences of underweight and stunted children were also significantly higher in rural than urban communities (19.4% vs. 9.3%, p < 0.001) and (43.3% vs. 12.6%, p < 0.001) respectively. In rural communities, the risk of stunted mothers having children with stunting was about 7 times higher than those who were not (OR 6.7, 95% CI = 1.4-32.0, p = 0.007). In urban communities, undernourished mothers have about 11 and 12 times risk of having children with underweight and wasting respectively (OR 11.2, 95% CI = 1.4-86.5, p = 0.005) and (OR 12.3, 95% CI = 1.6-95.7, p = 0.003) respectively. The identified risk factors for maternal and child under nutrition differs across rural and urban communities.

**Conclusions:**

The prevalence of maternal and child under-nutrition is high in both communities although higher in rural communities. Efforts at reducing the vicious cycle of under-nutrition among mothers and children should concentrate on addressing risk factors specific for each community.

## Background

The nutritional status of a mother is important, both as an indicator of her overall health and as a predictor of pregnancy outcome for both mother and child [1,2]. Adequate maternal nutrition is needed for breastfeeding, recovery following the stress of pregnancy and labour, coping with child rearing and care and also preparation for future pregnancies [1]. A mother who was malnourished as a fetus, young child or adolescent is more likely to enter pregnancy stunted and malnourished. Her compromised nutritional status affects the health and nutrition of her own children. These intergenerational effects can be turned into a vicious cycle perpetuating malnutrition from one generation to the next [3].

The consequences of poor maternal nutritional status are reflected in high infant and maternal morbidity and mortality [4,5]. Globally, more than 3.5 million mothers and children under five years of age die unnecessarily each year due to the underlying cause of under-nutrition, and millions more are permanently disabled by the physical and mental effects of poor nutrition, the vast majority in south-central Asia and sub-Saharan Africa [5].

The United Nations provided eight Millennium Development Goals (MDG), which serve as a framework for the entire international community to work together to reduce child morbidity and mortality [6]. These goals were adopted by all world leaders in 2000 and are set to be achieved by 2015. In Nigeria, an evaluation of the MDG between 2000 and 2007 showed that their achievement in reducing child mortality rate was unsatisfactory, while the maternal mortality rate was actually increasing [7]. Mid-way to the end of the MDG’s implementation period, there remain wide gaps between targets and actual achievement particularly in the rural communities. It is a well-known fact that, attainment of many of these MDG’s requires good nutrition status for children and adolescent girls.

Nigeria is the most populous country in Africa, with a population of about 148 million. About sixty percent of this population resides in the rural areas, while about forty-four percent of the population is made up of children and mothers [8]. In Lagos state, Nigeria, the infant mortality rate is 85 deaths per 1000 live births, relatively better than the national average. The maternal mortality rate is 650 deaths per 100,000 live births [9]. Most of these deaths have malnutrition as the predisposing factor, either directly or indirectly. However, these deaths are preventable. The risk factors for under-nutrition among mothers and children have been well described but data establishing differences in risk factors for under-nutrition between urban and rural communities for both mothers and children are few.

Therefore, this study was carried out to determine the relationship between the nutritional status of mothers and their children and the risk factors for under-nutrition among mothers and children living in rural and urban communities of Lagos state, Nigeria.

## Methods

### Study area

The study was carried out in the Epe Local Government Area and the Alimosho Local Government Area in Lagos State, Nigeria, which were randomly selected from the 20 local governments in Lagos state officially recognized by the Federal government of Nigeria.

Lagos state is Nigeria’s former capital city. The vegetation is that of the tropical rain forest. It has an annual rainfall of 180 centimeters and a mean temperature of 26°C. It is 200 – 500 meters above sea level. Lagos has a very diverse and fast-growing population, resulting from heavy and ongoing migration to the city from all parts of Nigeria as well as neighbouring countries. Though the Yoruba, an African people inhabiting southwest Nigeria, constitute the city’s principal ethnic group, there are a significant proportion of other ethnic groups, particularly the Ibos, Ibibio and Hausa.

### Study design

The study was a cross sectional survey of women in the reproductive age group and their children aged zero to fifty-nine months, in rural and urban communities located in randomly selected Local Government Areas of Lagos State, Nigeria.

### Ethical clearance

Ethical clearance was obtained from the Lagos State University Teaching Hospital Research/Ethics Committee; permission was also obtained from the parents that were involved.

### Method of sampling

A multistage random sampling technique was used to select subjects. Epe and Agbowa were randomly selected from the Epe LGA, representing urban and rural communities, respectively, while Akowonjo and Orisumbare were selected from the Alimosho LGA, representing urban and rural communities, respectively. From each communities, houses were selected ramdomly using the table of random numbers.

The sample size: The minimum number of the subjects ‘n’ required for the study was estimated from the formula:

$$n=z^{2}p\left(1–p\right)÷d^{2}$$

Where: ‘z’ is the critical value and in a two-tailed test it is equal to 1.96.

‘p’ is the estimated prevalence of malnutrition in women, which was taken as 9 percent, based on an earlier report by Nigerian Food Consumption and Nutrition Survey for Southwestern part of Nigeria [10].

‘d’ is the absolute sampling error that can be tolerated. In this study, it was fixed at 5 percent.

Therefore the minimum sample size: ‘ *n* ’ = 1.96^2^ × 0.09 × (1–0.09) ÷ 0.05^2^ = 125.9 which is approximately 126. Taking into consideration a possible attrition rate of 10 percent, the minimum sample size for this study will be 137.

This was rounded up to 150 each for rural and urban communities.

In each household, the parents of children aged zero to fifty-nine months were identified, and informed consent was obtained prior to the beginning of the study. The parents were interviewed according to the proforma specifically designed for the study. Information was obtained on demographic, socio-economic and environmental characteristics of the family. The families were assigned a socio-economic class using the method recommended by Ogunlesi *et al *[11]. The parents’ occupations and highest education attained were scored from 1 (highest) to 5 (lowest). The mean score for both parents gave the social class, falling within the 1 – 5 range. Those with mean scores of 1 and 2 were further classified as upper class, those with mean score of 3 were classified as middle class, while those with mean scores of 4 and 5 were classified as falling in the lower social class.

### Anthropometry

The children were weighed using an electronic weighing scale calibrated in 100 g units (SECA/UNICEF, Australia). Children who were too scared to stand on the scale were weighed together with the mother, and the mother’s weight automatically deducted to obtain the weight of the child. All children were weighed naked and to the nearest 0.1 kg.

Length of children aged less than twenty-four months was measured using an infantiometer. This was done on a firm surface with assistance, usually by the mother. The knees were held down and the head held firmly against the headboard. These measurements were done to the nearest 0.1 cm. Height was measured using a height board for children aged twenty-four to fifty-nine months. This was done with the children standing erect without shoes, with eyes facing forward and the feet together on the horizontal plane.

The mother’s weight was measured using the same weighing scale used for children.

The height of the mothers was also measured using a height board. This was done with the mothers standing erect, without shoes, with eyes facing forward and feet together on the horizontal plane. Standardization checks on the tools for anthropometric measurements were done periodically.

Malnutrition in children was calculated from the degree of stunting (height-for-age), underweight (weight-for-age) and wasting (weight-for-height) following World Health Organization (WHO) guidelines and cut off points [12]. In this study, a child was said to be underweight, wasted and stunted if the Weight-for-Age, Weight-for-Height and Height-for-Age were below minus two Standard Deviation (-2 SD) from the median of each international reference standard, respectively.

A mother was said to be undernourished if her BMI was less than 18.5 Kg/m^2^, or stunted if her height was less than 152 cm [12].

### Statistical analysis

WHO Anthro 2007 was used to generate z-score values for weight-for-age, height-for-age and weight-for-height. All data were entered into and analysed using SPSS for windows software version 13. The means and standard deviations (SD) were calculated for continuous variables, while ratios and proportions were calculated for categorical variables. Categorical variables were compared using the Pearson Chi square (*χ*2) test. Independent *t*-test was used to calculate mean differences for continuous variables between urban and rural communities. Using logistic regression, odds ratio and its 95% confidence interval were used to calculate risk of occurence of under-nutrition in a child if the mother is undernourished. ‘P’ values less than 0.05 were accepted as statistically significant.

## Results

### Socio-demographic characteristics

A total of 300 mother-child pairs were studied, consisting of 150 each from rural and urban communities, and the results obtained were analysed. Table 1 shows the socio-demographic characteristics and nutritional status of the study population according to their place of residence.

**Table 1:** Socio-demographic characteristics and nutritional status of study population according to place of residence

**Parameters**	**Total (n = 300)**	**Rural (n = 150)**	**Urban (n = 150)**	**P value**
**Mothers characteristics**				
Age (years)*	30.0 ± 5.6	29.6 ± 6.1	30.4 ± 5.6	0.186
**Family setting**				0.001
Monogamy	265 (88.3)	119 (79.3)	146 (97.3)	
Polygamy	35 (11.7)	31 20.7)	4 (2.7)	
**Parity**				0.001
1	83 (27.7)	24 (16.0)	59 (39.3)	
2	183 (61.0)	101 (67.3)	82 (54.7)	
5 – 7	34 (11.3)	25 (16.7)	9 (6.0)	
**Age at first birth**				0.001
15 – 19	29 (9.7)	22 (14.7)	7 (4.7)	
20 – 24	127 (42.3)	77 (51.3)	50 (33.6)	
25 – 29	96 (32.0)	41 (27.3)	55 (36.9)	
30 – 34	37 (12.3)	5 (3.3)	32 (21.3)	
35 - 39	8 (2.7)	3 (2.0)	5 (3.4)	
**Educational level of mother**				0.001
No formal education	4 (1.3)	3 (2.0)	1 (0.7)	
Primary	72 (24.0)	55 (36.7)	17 (11.3)	
Secondary	155 (51.7)	79 (52.7)	76 (50.7)	
Postsecondary	69 (23.0)	13 (8.7)	56 (37.3)	
**Social class**^**♯**^				0.001
Upper	19 (6.3)	0 (0.0)	19 (12.7)	
Middle	50 (16.7)	15 (10.0)	35 (23.3)	
Lower	231 (77.0)	135 (90.0)	96 (64.0)	
**Height, less than 152 cm**	27 (9.0)	11 (7.3)	16 (10.7)	0.313
**BMI (kg/m**^**2**^)*	24.9 ± 5.0	24.4 ± 5.0	29.4 ± 4.9	0.092
**Undernutrition, BMI < 18.5 kg/m**^**2**^	20 (6.7)	16 (10.7)	4 (2.7)	0.014^†^
**Children characteristics**				
Age (months)*	25.3 ± 16.4	31.3 ± 16.4	19.3 ± 4.0	0.001
**Sex**				0.248
Male	142 (47.3)	66 (44.0)	76 (50.7)	
Female	158 (52.7)	84 (56.0)	74 (49.3)	
**Birth order**				0.000
1	118 (39.3)	39 (26.0)	79 (52.7)	
2-4	161 (53.6)	97 (64.7)	64 (42.7)	
5 – 7	21 (7.0)	14 (9.3)	7 (4.7)	
**Weight-for-age z score, < -2 SD**	43 (14.3)	29 (19.4)	14 (9.3)	0.001
**Height-for-Age z score, < -2 SD**	84 (28.0)	65 (43.3)	19 (12.6)	0.001
**Weight-for-height z score, < -2 SD**	31 (8.3)	18 (12.0)	13 (8.7)	0.118

Values are number (%) unless otherwise stated; *values are mean ± SD; ^†^Adjusted for social class. ^♯^Upper social class includes parents such as senior government employee, high scale traders, and professionals, middle class include junior government employee, teachers and technicians while lower social class are peasant farmers, artisans, security agents, messengers, apprentice, laborers and the unemployed.

Age at first birth of mothers in rural communities was significantly lower than for mothers in urban communities (p < 0.001). Polygamous homes was siginificantly common in rural than uban communities (p < 0.001) while the parity of mothers was higher in rural than urban communities (p < 0.001). The educational level of mothers was significantly better in urban as opposed to rural areas (p < 0.001) and a significant proportion of the mothers in rural compared with urban communities (90.0% vs. 60.4%, p < 0.001) were from the lower social class.

The mean age of the children in the rural communities was significantly higher than that of children from urban communities (31.3 vs. 19.3 months; p < 0.001). Gender distributions of the children were not statistically different, with a male: female ratio of 1:1.3 in rural areas and 1:1 in urban areas. Seventy-nine (52.7%) children in urban areas were the first born compared with 39 (26%) children in rural areas. This difference was statistically significant (p < 0.001).

### Nutritional status of mothers and children

Using a BMI of 18.5 kg/m^2^ as the cut off point, 16 (10.7%) mothers from rural communities and 4 (2.7%) mothers from urban communities were undernourished. This difference was significant even after adjusting for social class (OR 4.3, 95% CI = 1.4-13.4, p = 0.014). However, there was no significant difference in the pevalence of stunting between mothers from rural and urban communities (7.3% vs. 10.7%, p = 0.313) respectively.

The prevalences of underweight and stunting were significantly higher among children from rural than urban areas (19.4% vs. 9.3%, OR 3.8, 95% CI = 1.8-8.1, p < 0.001) and (43.3% vs. 12.6%, OR 7.4, 95% CI = 3.8-14.1, p < 0.001) respectively.

### Relationship between maternal and child nutritional status

Table 2 shows the relationship between stunted mothers and the nutritional status of children. In rural communities, the risk of stunted mothers having chidren with stunting is about 7 times higher than mothers that are not stunted (OR 6.7, 95% CI = 1.4-32, p = 0.007).

**Table 2:** **Relationship between mother’s height and child’s nutritional status**^**†**^

**Rural**				
**Indicator of child**	**Mother’s height (cm)**		**Odds ratio**	**95% CI**
**Nutritional status**	**< 152**	**≥ 152**		
**Weight-for-age Z-score**				
Underweight	3 (10.7)	26 (89.3)	1.63	0.40 – 6.57
Not underweight	8 (6.6)	113 (93.4)	1	
**Height-for-age Z-score**				
Stunted	9 (13.9)	56 (86.1)	6.67*	1.39 – 32.0
Not stunted	2 (2.4)	83 (97.6)	1	
**Weight-for-height Z-score**				
Wasted	2 (11.1)	16 (88.9)	1.71	0.34 – 8.62
Not wasted	9 (6.8)	123 (93.2)	1	
**Urban**				
**Weight-for-age Z-score**				
Underweight	2 (14.3)	12 (85.7)	1.45	0.29 – 7.16
Not underweight	14 (10.3)	122 (89.7)	1	
**Height-for-age Z-score**				
Stunted	3 (15.8)	16 (84.2)	1.70	0.44 – 6.63
Not stunted	13 (9.9)	118 (90.1)	1	
**Weight-for-height Z-score**				
Wasted	0 (0.0)	13 (100)	undefined	
Not wasted	16 (11.7)	121 (88.3)		

*p < 0.01; ^†^Adjusted for social class, age at first birth, parity and family setting.

Table 3 shows the relationship between under-nutrition in mothers and the nutritional status of children. In urban communities, the risk of undernourished mothers having childen with underweight and wasting were about 11 and 12 times respectively higher than mothers that were not undernourished (OR 11.2, 95% CI = 1.4-86.5, p = 0.005) and (OR 12.3, 95% CI = 1.6-95.7, p = 0.003) respectively. Although, the risk of undernourished urban mothers having stunted children was about 2 times higher than those not undernourished, this difference was not significant (OR 2.4, 95% CI = 0.2-24, p = 0.452).

**Table 3:** **Relationship between mother and child nutritional status**^**†**^

**Rural**				
**Indicator of child**	**Mother’s BMI (kg/m**^**2**^**)**		**Odds ratio**	**95% CI**
**Nutritional status**	**< 18.5**	**≥ 18.5**		
**Weight-for-age Z-score**				
Underweight	1 (3.4)	28 (96.6)	0.25	0.03- 1.99
Not underweight	15 (12.4)	106 (87.6)	1	
**Height-for-age Z-score**				
Stunted	8 (12.3)	57 (87.7)	1.35	0.48 – 3.81
Not stunted	8 (9.6)	77 (90.4)	1	
**Weight-for-height Z-score**				
Wasted	3 (16.7)	15 (83.3)	1.83	0.47- 7.17
Not wasted	13 (10.0)	119 (90.0)	1	
**Urban**				
**Weight-for-age Z-score**				
Underweight	2 (14.3)	12 (85.7)	11.2*	1.44 – 86.5
Not underweight	2 (1.5)	134 (98.5)	1	
**Height-for-age Z-score**				
Stunted	1 (5.3)	18 (94.7)	2.37	0.23 – 24.03
Not stunted	3 (2.3)	128 (97.7)	1	
**Weight-for-height Z-score**				
Wasted	2 (15.4)	11 (84.6)	12.3*	1.57 – 95.7
Not wasted	2 (1.5)	135 (98.5)	1	

*P < 0.01; ^†^Adjusted for social class, age at first birth, parity and family setting.

### Risk factors for under-nutrition among mothers

Tables 4 and 5 shows the influence of various socio-demographic factors on the nutritional status of mothers in rural and urban communities. In rural communities, there was significant association between nutritional status of mothers and family setting (*χ*^2^ = 4.67, p = 0.031), age at first birth (*χ*^2^ = 12.2, p = 0.016), and level of education of mothers (*χ*^2^ = 34.7, p < 0.001). While in urban communities, nutritional status of mothers was significantly related to parity (*χ*^2^ = 28.2, p < 0.001) and level of education of mothers (*χ*^2^ = 15.4, p < 0.001).

**Table 4:** Nutritional status in relation to socio-demographic variables for rural mothers

**Parameters**	**Number of women (n =150)**	**Number (%) malnourished**	**P values**
**Age (years)**			0.090
15 – 19	1	0 (0.0)	
20 – 24	25	2 (8.0)	
25 – 29	56	11 (19.6)	
30 – 34	36	2 (5.6)	
35 – 49	32	1 (3.1)	
**Marital status**			0.782
Not yet married	3	0 (0.0)	
Married	146	16 (11.0)	
Widow	1	0 (0.0)	
**Family setting**			0.031
Monogamy	119	16 (13.4)	
Polygamy	31	0 (0.0)	
**Age at first birth**			0.016
15- 19	22	0 (0.0)	
20 – 24	78	14 (17.9)	
25 – 29	42	1 (2.4)	
30 – 34	5	0 (0.0)	
35 - 39	3	1 (33.3)	
**Parity**			0.587
1	24	3 (12.5)	
2	51	8 (15.7)	
3	22	0 (0.0)	
4	28	3 (10.7)	
5 -7	25	2 (8.0)	
**Education of mothers**			0.000
No formal education	3	0 (0.0)	
Primary	55	1 (1.8)	
Secondary	79	15 (19.0)	
Post-secondary	13	0 (0.0)	
**Social class**			0.514
Upper	0	0 (0.0)	
Middle	15	1 (6.7)	
Lower	135	15 (11.1)	

**Table 5:** Nutritional status in relation to socio-demographic variables for urban mothers

**Parameters**	**Number of women (n = 150)**	**Number (%) malnourished**	**P values**
**Age (years)**			0.533
15 – 19	0	0 (0.0)	
20 – 24	18	1 (5.6)	
25 – 29	52	1 (1.9)	
30 – 34	35	0 (0.0)	
35 – 49	45	2 (4.4)	
**Marital status**			0.478
Not yet married	3	0 (0.0)	
Married	147	4 (2.7)	
**Family setting**			0.737
Monogamy	146	4 (2.7)	
Polygamy	4	0 (0.0)	
**Age at first birth**			0.130
15- 19	7	1 (14.3)	
20 – 24	50	2 (4.0)	
25 – 29	55	1 (1.8)	
30 – 34	32	0 (0.0)	
35 - 39	5	0 (0.0)	
**Parity**			0.000
1	59	1 (1.7)	
2	49	1 (2.0)	
3	23	0 (0.0)	
4	10	0 (0.0)	
5 - 6	9	2 (22.2)	
**Education of mothers**			0.000
No formal education	1	0 (0.0)	
Primary	17	3 (17.6)	
Secondary	76	1 (1.3)	
Post-secondary	56	0 (0.0)	
**Social class**			0.128
Upper	19	0 (0.0)	
Middle	35	0 (0.0)	
Lower	96	4 (4.4)	

### Risk factors for under-nutrition among children

Tables 6 and 7 shows the influence of various socio-demographic factors on the nutritional status of children in rural and urban communities. In rural communities, Stunting was significantly related to lower parents’ social class (*χ*2 = 10.5, p = 0.005) only while wasting was significantly associated with low age of child (*χ*^2^ = 15.0, p = 0.005) and low educational level of the father (*χ*^2^ = 22.8, p = 0.03). In urban communities, underweight was significantly related to gender (*χ*^2^ = 4.81, p = 0.028) and birth order (*χ*^2^ = 16.5, p = 0.006). Stunting was significantly related to gender (*χ*^2^ = 7.0, p = 0.008) only while wasting was significantly related to birth order (*χ*^2^ = 17.5, p = 0.004) only.

**Table 6:** Nutritional status in relation to socio-demographic variables for rural children

**Parameters**	**Number of children (No = 150)**	**≤ -2 SD WAZ**		**≤ -2 SD HAZ**		**≤ -2 SD WHZ**	
		**No (%)**	**P value**	**No (%)**	**P value**	**No (%)**	**P value**
**Age (months)**			0.085		0.693		0.012
0 – 11	11	4 (36.4)		4 (36.4)		4 (36.4)	
12 – 23	42	13 (31.0)		22 (52.4)		7 (16.7)	
24- 35	34	2 (5.9)		17 (50.0)		0 (0.0)	
36 – 47	28	4 (14.3)		7 (25.0)		1 (3.6)	
48 – 59	35	5 (14.3)		13 (37.1)		4 (11.4)	
**Sex**			0.187		0.026		0.238
Male	66	16 (24.2)		27 (40.9)		8 (12.1)	
Female	84	12 (14.2)		36 (42.9)		8 (9.5)	
**Birth order**			0.365		0.241		0.767
1	38	10 (26.3)		14 (36.8)		7 (18.4)	
2	45	5 (11.1)		23 (51.1)		3 (6.7)	
3	33	6 (18.1)		13 (39.4)		3 (9.1)	
4	19	6 (31.5)		10 (52.6)		1 (5.3)	
5 – 7	14	1 (7.1)		3 (21.4)		2 (14.2)	
**Total number of children**			0.070		0.078		0.947
1	23	9 (39.1)		10 (43.5)		4 (17.4)	
2	50	6 (12.0)		21 (42.0)		6 (12.0)	
3	28	3 (10.7)		16 (57.1)		2 (7.1)	
4	25	4 (16.0)		6 (24.0)		2 (8.0)	
≥ 5	23	6 (26.1)		10 (43.5)		2 (8.7)	
**Educational level of mother**			0.706		0.066		0.741
No formal education	3	2 (66.7)		2 (66.7)		1 (33.3)	
Primary	55	11 (20.0)		26 (47.3)		7 (12.7)	
Secondary	79	14 (17.7)		32 (40.5)		7 (8.9)	
Postsecondary	13	2 (15.4)		3 (23.1)		1 (7.7)	
**Educational level of father**			0.378		0.142		0.000
No formal education	4	2 (50.0)		3 (75.0)		2 (50.0)	
Primary	13	3 (23.1)		5 (38.5)		2 (15.4)	
Secondary	99	18 (18.2)		49 (49.5)		9 (9.1)	
Postsecondary	34	7 (14.6)		6 (17.6)		5 (8.8)	
**Social class**			0.330		0.005		0.352
Upper	0	0 (0.0)		0 (0.0)		0 (0.0)	
Middle	15	3 (30.0)		1 (6.7)		2 (13.3)	
Lower	135	25 (18.5)		62 (45.9)		14 (10.4)	

**Table 7:** Nutritional status in relation to socio-demographic variables for urban children

**Parameters**	**Number of children (No = 150)**	**≤ -2 SD WAZ No (%) P value**		**≤ -2 SD HAZ No (%) P value**		**≤ -2 SD WHZ No (%) P value**	
**Age (months)**			0.105		0.713		0.815
0 – 11	50	8 (16.0)		8 (16.0)		5 (10.0)	
12 – 23	48	6 (12.5)		8 (16.7)		6 (12.5)	
24- 35	27	0 (0.0)		2 (7.4)		2 (7.4)	
36 – 47	18	0 (0.0)		1 (5.6)		0 (0.0)	
48 – 59	7	0 (0.0)		0 (0.0)		0 (0.0)	
**Sex**			0.028		0.008		0.810
Male	76	11 (14.5)		15 (19.7)		7 (9.2)	
Female	74	3 (4.1)		4 (5.4)		6 (8.1)	
**Birth order**			0.006		0.653		0.004
1	79	6 (7.6)		12 (15.2)		7 (8.9)	
2	34	4 (11.8)		7 (20.6)		2 (5.9)	
v3	21	0 (0.0)		0 (0.0)		0 (0.0)	
4	9	2 (22.2)		0 (0.0)		2 (22.2)	
≥ 5	7	2 (28.6)		0 (0.0)		2 (28.6)	
**Total number of children**			0.109		0.109		0.094
1	59	8 (13.6)		11 (18.6)		5 (8.5)	
2	48	2 (4.2)		8 (16.7)		4 (8.3)	
3	22	0 (0.0)		0 (0.0)		0 (0.0)	
4	12	2 (16.7)		0 (0.0)		2 (16.6)	
≥ 5	9	2 (22.2)		0 (0.0)		2 (22.2)	
**Educational level of mother**			0.782		0.402		0.832
No formal education	1	0 (0.0)		0 (0.0)		0 (0.0)	
Primary	17	2 (11.8)		1 (5.9)		2 (11.8)	
Secondary	76	10 (13.2)		11 (14.5)		6 (7.9)	
Postsecondary	56	2 (3.6)		7 (12.5)		5 (8.9)	
**Educational level of father**			0.910		0.541		0.953
No formal education	1	0 (0.0)		1 (100.0)		0 (0.0)	
Primary	7	2 (28.6)		1 (14.3)		2 (28.6)	
Secondary	69	6 (8.7)		8 (11.6)		5 (7.2)	
Postsecondary	73	6 (8.2)		9 (12.3)		6 (8.2)	
**Social class**			0.197		0.519		0.472
Upper	19	1 (5.2)		1 (5.2)		0 (0.0)	
Middle	35	1 (18.5)		4 (11.4)		5 (14.3)	
Lower	96	12 (12.5)		14 (14.6)		8 (8.3)	

## Discussion

This study has shown that the prevalence of maternal and child under-nutrition among mothers and children in Lagos state, Nigeria remains high particularly in the rural communities. This is consistent with pevious studies on Nigerian women and children [13,14]. In our study, many of the socio-demographic factors which favour the occurrence of maternal and child under-nutrition were significantly higher in the rural communities. For instance, teenage pregnancy and high parity which are known to deplete mothers of nutrients because of physiological immaturity and the stresses of multiple pregnancies and lactations with inadequate recuperative intervals between pregnancies were commoner in rural communities [15]. Similarly, low maternal education and low socio-economic class which limit mothers’ utilisation of available resouces for the improvement of their own nutritional status and that of their families were also commoner in rural communities. These may explain the high prevalence of maternal and child under-nutrition in our rural communities.

The overall prevalence of under-nutrition for mothers in this study was 6.7%; this is within the range of 5 to 20% reported for African women [16]. However, it is lower than the 25% reported for Ethiopian women [17], over 60% for Indian women [18] and 43.7% for women in Bangladesh [19]. This difference might be a reflection of the different environmental, cultural and socioeconomic situations in these countries which further implicates the important role of poverty, ignorance, food insecurity and infectious disease burden in maternal and child under-nutrition.

Despite several approaches and attempts at reducing rates of under-nutrition among Nigerian children, the prevalence remains high. In response to this problem of malnutrition, the Nigerian government launched its national policy on food and nutrition in 2002, with the goal of improving the nutritional status of all Nigerians [20]. This policy sets specific targets, which include reducing moderate and severe malnutrition among children under five years old by 30% by the year 2010. Instead of a reduction, what has been observed is that the national prevalence of under-nutrition remained largely stable between the years 2003 and 2009, with the prevalences of underweight, wasting and stunting essentially static at 24%, 11% and 43%, respectively [21]. In our study, the values from rural areas were similar to these national figures, while the values from urban areas were lower than the national figures, except for the prevalence of wasting. This implies that efforts at achieving effective control of under-nutrition should be concentrated in rural areas.

In both rural and urban communities, we observed a relationship between mother and child under-nutrition. This is similar to what was observed in rural Guinea by Mock et al [22] and in rural Ethiopia by Lindtjorn and Alemu [23]. In particular, in the rural communities, there was an association between chronic malnutrition in mothers and their children. This could be due to the influence of genetics on height, or could actually be due to a vicious circle of poorly nourished female children who grow up to be stunted and then bear their own children who are equally under-nourished, continuing the cycle. It is worth noting that differences exist in the maternal-child nutrition associations across rural and urban communities. In rural communities, the maternal child nutrition association was chronic malnutrition while in urban it was acute malnutrition and this raises suspicion of persistent suboptimal nutritional practices in rural communities. Malnutrition has short and long term consequences on health of children. An important long term consequence of chronic malnutrition is that children with stunting are prone to accumulation of body fat, especially central fat [24]. This could increase the burden of non-communicable diseases such as diabetes mellitus and hypertension thereby further stretching the health system.

In this study, the identified risk factors for maternal under-nutrition were different across rural and urban communities. The only risk factor that cut across both communities was low maternal education. In the rural communities, the lower prevalence of maternal under-nutrition in polygamous homes was suggestive of supportive role of co-wives for each other and possibly their husband. According to Madhaven [25], it is also possible for the co-wives to live in competition with each other resulting in poor maternal and child health. Similar to finding in other studies [16,17], mothers in our rural communities who started having children at a low age and those who started late were at risk of poor nutritional status. This has been ascribed to physiological unpreparedness and maternal depletion of nutrients. In our urban communities, mothers having more than 4 children were at risk of under-nutrition and this is also similar to the findings in other studies [17,26]. This is instructive, if one considers the fact that the standard of living in Lagos city is high and having more children ordinarily translate to more stresses of pregnancy, more financial burden, little food to eat and therefore poor maternal nutritional status. For children, the risk factors for underweight, wasting and stunting also differs across rural and urban communities. In rural communities, they were mostly socio-economic factors while in urban communities they were mostly biological factors. These risk factors need to be taking into consideration when planning interventional strategies aimed at reducing maternal and child under-nutrition in our rural and urban communities.

Though our study was limited to 2 local government areas of Lagos State, the findings can be generalized to the broader community. We recommend that further cross-sectional research work involving other communities and a larger sample frame is needed to better understand the relationship between mother and child under-nutrition and the risk factors for under-nutrition among mothers and children.

## Conclusions

In conclusion, this study reveals high prevalence of maternal and child under-nutrition in both communities particularly in rural communities. It also confirms relationship between maternal and child under-nutrition. The risk factors for maternal and child under-nutrition differs across rural and urban communities. In order to reduce morbidity and mortality among mothers and children to the barest minimum, every effort must be made by the government to improve maternal and child nutritional status. This may involve public health enlightenment campaign discouraging teenage pregnancy and high parity of mothers which predisposes to poor nutritional status, complications during pregnancy and therefore risk of poor outcomes both for the mother and the child. There is also need to increase women education as this will make them receptive to health interventions that will improve their nutritional status and that of their child. It will also improve their earning power and hence good food puchasing power for the household.

## Competing interests

The authors declare that they have no competing interest.

## Authors’ contributions

IOS concieved, designed, analyzed the data and wrote the first draft. IOO and WAO participated in the design, supervised data collection and guided writing of the manuscript. COS participated in the design and data collection. All authors read and approved the final manuscript.
